# Exploring *Aegilops caudata*: A Comprehensive Study of the *CslF6* Gene and β-Glucan

**DOI:** 10.3390/genes15020168

**Published:** 2024-01-27

**Authors:** Ilaria Marcotuli, Davide Caranfa, Pasqualina Colasuonno, Stefania Lucia Giove, Agata Gadaleta

**Affiliations:** Department of Soil, Plant and Food Sciences, University of Bari Aldo Moro, Via G. Amendola 165/A, 70126 Bari, Italy; davide.caranfa@uniba.it (D.C.); pattybiotec@yahoo.it (P.C.); stefanialucia.giove@uniba.it (S.L.G.); agata.gadaleta@uniba.it (A.G.)

**Keywords:** *Aegilops caudata*, *CslF6* gene, comparative genomics, β-glucan biosynthesis

## Abstract

In the quest for sustainable and nutritious food sources, exploration of ancient grains and wild relatives of cultivated cereals has gained attention. *Aegilops caudata*, a wild wheatgrass species, stands out as a promising genetic resource due to its potential for crop enhancement and intriguing nutritional properties. This manuscript investigates the *CslF6* gene sequence and protein structure of *Aegilops caudata*, employing comparative analysis with other grass species to identify potential differences impacting β-glucan content. The study involves comprehensive isolation and characterization of the *CslF6* gene in *Ae. caudata*, utilizing genomic sequence analysis, protein structure prediction, and comparative genomics. Comparisons with sequences from diverse monocots reveal evolutionary relationships, highlighting high identities with wheat genomes. Specific amino acid motifs in the CslF6 enzyme sequence, particularly those proximal to key catalytic motifs, exhibit variations among monocot species. These differences likely contribute to alterations in β-glucan composition, notably impacting the DP3:DP4 ratio, which is crucial for understanding and modulating the final β-glucan content. The study positions *Ae. caudata* uniquely within the evolutionary landscape of *CslF6* among monocots, suggesting potential genetic divergence or unique functional adaptations within this species. Overall, this investigation enriches our understanding of β-glucan biosynthesis, shedding light on the role of specific amino acid residues in modulating enzymatic activity and polysaccharide composition.

## 1. Introduction

In the pursuit of sustainable and nutritious food sources, researchers have turned their attention to ancient grains and wild relatives of cultivated cereals [1]. Among these, *Ae. caudata*, a wild wheatgrass species, has gained prominence due to its potential as a valuable genetic resource for crop improvement and its intriguing nutritional properties. In particular, the seeds of *Ae. caudata* have been a subject of increasing interest for their unique nutrient content and their association with genes responsible for β-glucan biosynthesis [2]. 

*Ae. caudata* belongs to the genus *Aegilops* within the grass family *Poaceae*. It is native to regions of the Middle East and is closely related to cultivated wheat species (*Triticum* spp.). Unlike cultivated wheat, *Ae. caudata* has not undergone extensive domestication, making it a valuable genetic resource for breeding programs aimed at improving the resilience, disease resistance, and nutritional content of wheat and related cereals. Its genetic diversity and adaptation to various environmental conditions make it an intriguing subject for research in the context of sustainable agriculture [3,4].

*Ae. caudata* seeds have garnered attention for their unique nutrient composition. These seeds are a rich source of essential nutrients, including proteins, vitamins, minerals, and dietary fiber. The protein content of *Ae. caudata* seeds is of particular interest, as it contains a balanced amino acid profile, including essential amino acids. Furthermore, these seeds are known to be rich in micronutrients such as iron, zinc, and folate, which are critical for human health. The dietary fiber content, including β-glucans, in *Ae. caudata* seeds further adds to their nutritional appeal [5,6]. 

B-glucans are a group of polysaccharides found in the cell walls of various cereal grains, including wheat, oats, and barley [7,8,9]. They are known for their potential health benefits, particularly lowering cholesterol levels and supporting digestive health [10,11,12]. The molecular structure of β-glucan dictates its function. According to current understanding, β-1,3/1,4 glycosidic bonds link cereal β-glucans. In the structure of cereal β-glucan, the β-1,4-linked glucose chain is interspersed with β-1,3 linkages. While trimers and tetramers are the most common consecutive β-1,4 segments, longer cellulose-like segments are also present in β-glucan molecules [13]. Significantly, two primary oligomer units, DP3 and DP4, account for over 90% of cereal β-glucan structures, varying among species and between genotypes [14].

Understanding the genetic basis of β-glucan biosynthesis is crucial for crop improvement and the development of cereal varieties with enhanced β-glucan content [7].

In recent years, significant progress has been made in identifying and characterizing the genes involved in β-glucan biosynthesis in various cereal species [7,10,15,16]. In the case of *Ae. caudata*, research efforts have focused on elucidating the genetic mechanisms responsible for the synthesis of β-glucans in its seeds. These insights can potentially lead to the development of wheat varieties with improved β-glucan content through breeding or genetic modification techniques [6].

B-glucans exhibit diverse physiological and nutritional functions in plants. They are vital components of cell walls, where they contribute to structural integrity and resistance against environmental stresses [17,18]. Moreover, β-glucans function as storage polysaccharides in various plant organs, such as seeds, grains, and tubers, serving as an energy reserve during germination and growth [19,20].

Our knowledge regarding how and where β-glucans are produced remains largely incomplete, as well as which genes are involved, their functions, and interactions, and the specific activities of the enzymes. A group of genes, forming a superfamily, plays a significant role in the synthesis of these polysaccharides. This superfamily comprises the *cellulose synthase* (*Ces*) [21,22,23] and *cellulose-synthase-like* (*Csl*) [24] families.

The *Csl* superfamily is responsible for synthesizing several plant cell wall polysaccharides, organized into subfamilies labeled A to H, each of which consists of multiple genes [25]. For instance, in rice (*Oryza sativa* L.), there are a total of 37 *Csl* genes [26], whereas *Arabidopsis* has 30 [27]. Notably, not all *Csl* subfamilies are represented in all higher plant groups. The *CslB* and *CslG* subfamilies are exclusive to dicotyledons and gymnosperms, while the *CslF* and *CslH* groups are only found in monocotyledons [28]. These subfamilies directly or indirectly regulate the abundance and fine structure of β-glucans in both grain and other parts of the plant [29,30,31].

Research conducted by Burton et al. [32] revealed that over-expressing a *CslF* gene, under the control of an endosperm-specific promoter, led to an increase in β-glucan content and a significant reduction in starch in transgenic grains. Given that the β-glucan and starch pathways compete for the initial substrate—glucose—used in their synthesis, *Brachypodium distachyon*, with over 40% of its grain weight as β-glucan and only about 6% starch, further supports a regulatory connection between starch and β-glucan synthesis [29]. Additionally, when *Arabidopsis* was transformed with the *OsCslF6* gene, it produced mixed-linkage glucan in the cell wall, indicating the capability of *CslF6* to synthesize β-glucan [33]. In barley, four corresponding *CslF* genes were mapped to chromosome 2H (*HvCslF3*, *HvCslF4*, *HvCslF8*, *HvCslF10*), with two other genes on chromosomes 1H (*HvCslF9*) and 7H (*HvCslF6*), corresponding to quantitative trait loci (QTL) for grain β-glucan content [34,35].

In rice, knockout mutants of *OsCslF6* synthesize minimal β-glucan content. Nemeth et al. [36] identified the *CslF6* gene in wheat and demonstrated that transgenic manipulation through iRNA could modify the amounts and properties of β-glucan in wheat. Other studies showed that the addition of barley chromosome 7H (where *HvCslF6* is located) to the wheat genome increases β-glucan production [37]. The wheat–barley addition lines, obtained through hybridization, contain a genetic background of common wheat, allowing the genetic analysis of a single barley chromosome affecting the final phenotype. They have been used for several trait studies as the production of bioactive compounds [38,39,40].

In this scenario, *Ae. caudata* stands as a promising genetic resource with unique nutrient content and the potential to contribute to the enhancement of cereal crops. The investigation of genes involved in β-glucan biosynthesis in this species not only offers insights into its nutritional properties but also flags the way for improving the health-promoting aspects of cultivated wheat. This paper investigates the cellulose synthase F6 gene sequence and protein structure of *Ae. caudata* and carries out a comparative analysis with other grasses to identify possible differences among species and correlate them with the final β-glucan content.

## 2. Materials and Methods

### 2.1. Plant Material and DNA Extraction

The *Aegilops caudata* genotype from the GenBank of the Department of Soil, Plant and Food Sciences (University of Bari, Italy) was used to characterize the *CslF6* gene. The genotype was grown in Valenzano (Bari, Department of Soil, Plant and Food Science, University of Bari Aldo Moro), and leaves were harvested at tillering time. Genomic DNA was extracted from fresh leaves using the technique outlined in Sharp et al. [41] and subsequently underwent purification through phenol-chloroform extraction. The quality and concentration of the DNA were assessed via spectrophotometric analysis using the NanoDrop2000 (Thermo Scientific™, Thermo Fisher Scientific, Waltham, MA, USA 02451) at 260 and 280 nm, with an A260/A280 ratio falling within the range of 1.6 to 1.8, and checked by agarose gel-electrophoresis. 

For fragment sequencing, DNA amplifications were conducted in 25 mL reaction mixtures, with each mixture containing 25 ng of template DNA, 2 mM of each primer, 200 mM of each dNTP, 2.5 mM of MgCl2, 1X PCR buffer (10 mM TRIS-HCl, pH 8.3, 10 mM KCl), and 0.5 units of Taq DNA polymerase. The PCR protocol used in a Perkin Elmer DNA Thermal Cycler (Norwalk, CT, USA) was as follows: initial denaturation at 95 °C for 5 min, followed by 35 cycles of denaturation at 95 °C for 1 min, annealing at 55 °C/65 °C for 2 min, and extension at 72 °C for 1 min, with a final extension step at 72 °C for 15 min. The PCR products were visualized through 1.5% agarose gel electrophoresis.

### 2.2. Cellulose Synthase Gene (CslF6) Isolation and Characterization

To isolate the complete sequences of the *Ae. caudata CslF6* gene, we used the sequences of durum wheat, previously isolated by our lab [42] as an initial query probe to blast the Persephone^®^ multi-genome browser (https://web.persephonesoft.com/?data=genomes/TA1851, accessed on 8 November 2023), which contain the *Ae. umbellulata* genome assemblies. From the Persephone^®^ browser the sequence with 99% of similarity located on chromosome 7U (*chr7U_TA1851*) was used as a probe for the primer design. 

In order to obtain the entire gene sequences, a complete set of genome specific primer pairs were designed by using ‘Primer3 Input’ (version 0.4.0)’ software. Single PCR fragments were directly purified with an EuroGold Cycle Pure Kit and sequenced in both frame direction (5′->3′ and 3′->5′) using BigDye chemistry (Applied Biosystems) in a 96 capillary automatic sequencer ABI PRISM 3500. Gaps and uncertain sequence were resolved by primer walking. Regions of less coverage or ambiguous reads were rechecked with additional primers. Sequence assembly was obtained with ‘Codone Code Aligner’ (version 11.0.2 ) and ‘Geneious’ (version 2023.2.1) assembly programs.

Gene prediction was conducted with the FGENESH program (http://linux1.softberry.com/berry.phtml?topic=fgeneshandgroup=programsandsubgroup=gfind, accessed on 27 September 2023). Consensus exon/introns boundaries were confirmed using grass expressed sequence tag sequences aligned to the genomic sequence.

### 2.3. Aegilops caudata CslF6 Protein Sequence and Structure

To predict the protein sequence and structure based on the genomic sequence, Geneious software (version 2023.2.1) was employed for the sequence translation and prediction of transmembrane regions, coiled coil regions, conserved regions between wheat and barley proteins. Additionally, the *Ae. caudata* CslF6 protein newly obtained was aligned with the corresponding sequences from the alignment of the CslF6 from *Oryza sativa*, *Setaria italica*, *Sorghum bicolor*, *Zea mays*, *Brachypodium*, *Avena sativa*, *Hordeum vulgare*, wheat (A, B and D genomes), *Aegilops strangulata*, *Trititucm dicoccoides* and *Trititcum urartu*. Protein sequences were additionally employed in the phylogenetic examination using the neighbor joining method (NJ), and its topology was evaluated using 1000 bootstrap replicates implemented in ‘Geneious’. Differences in the residues of the principal motif of the proteins were investigated to elucidate the potential impact of particular amino acid variations and their spatial arrangements near the active site on the intricate structure of the (1,3;1,4)-β-glucan synthesized. Homology models with cellulose synthases of bacteria (BCSA) were also implemented using the sequences from rice, maize, sorgo and *Setaria*, *Triticum* subspecies, *Brachypodium*, oat, and barley.

### 2.4. Promoter Cis-Acting Element Distribution Analysis

The 2000 -bp sequences upstream of the start codon of *Ae. caudata CslF6* gene was extracted as the promoter region and submitted to the PlantCARE database (https://bioinformatics.psb.ugent.be/webtools/plantcare/html/, accessed on 11 January 2024) for prediction of the cis-acting elements. 

## 3. Results

### 3.1. Isolation and Characterization of Cellulose Synthase-like F6 Gene (CslF6) in Ae. caudata

**Figure 1:**
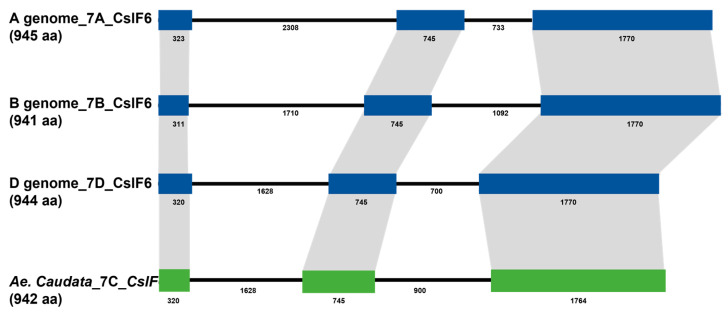
Comparison of gene structures between *Ae. caudata* and *Triticum* genome sequences (A, B and D) based on colored boxes, highlighting conserved exons. Intron and exon sizes are shown, as well as the whole gene (in brackets). The *CslF6* genes, in the genomes reported, are composed of three exons of conserved sizes and two introns.

The sequence corresponding to the *CslF6* gene from the durum wheat genomic sequences, previously isolated by our group [42], was used as a query blast to the *Ae. umbellulata* assembly deposit in the Persephone^®^ multi-genome browser to design specific primer pairs for the isolation of the *CslF6* full gene sequence in *Ae. caudata*. All the amplification analyses were carried out on DNA extracted from leaves of the *Ae. caudata* genotype grown in Valenzano (Bari, Italy). Through the genotype used for the analysis, the C genome sequence of *CslF6* was isolated with the corresponding cDNA. The genomic sequence was 5357 bp, including an mRNA of 2829 bp and a protein of 942 aa (Figure 1). Fgenesh++ (version 2.1) software was used for gene prediction to define the intron/exon structure, predicting a gene structure composed of three exons and two introns (Figure 1). Using Phytozome (version 13) software, a comparison between wheat and barley sequences was carried out through blast analysis. Considering the *Ae. caudata CslF6* with the wheat genome sequences, the identities detected were 96.8% with the A genome, 97.1% with the B genome, and 98.9% with the D genome, while considering the cDNA, the similarities were 97.4% with the A genome, 97.6% with the B genome, and 99.2% with the D genome. 

Using the newly obtained sequence, we were able to localize the *CslF6* gene on the *Ae. umbellulata* genome through the Persephone^®^ multi-genome browser page (https://web.persephonesoft.com/?data=genomes/TA1851, *accessed on 08/11/2023*). The gene was localized on chromosome chr7U_TA1851 at the physical position from 204.549.972 to 204.555.410 bp. Comparing the position of the gene with other species, the CslF6 was located on: chromosome 7H for barley (*HORVU7Hr1G070010*); chromosome group 7 for wheat (*TraesCS7A02G298600*, *TraesCS7B02G188400*, and *TraesCS7D02G294300* for bread wheat; *TRITD7Av1G149750* and *TRITD7Bv1G108090* for durum wheat) and *T. dicoccoides* (*TRIDC7AG041550* and *TRIDC7BG030910*); chromosome 7 for *Sorghum* (*SORBI_3007G050600*); chromosome group 7 for oat (*AVESA.00001b.r3.7Ag0002427*, *AVESA.00001b.r3.7Cg0002511*, *AVESA.00001b.r3.7Dg0001419*); chromosome 7 for *urartu* (*LOC125522276*); chromosome 7D for *Ae. strangulata* (*LOC109773098*); chromosome 6 for *S. italica* (*XM_004972717*); chromosome 10 for maize (*GRMZM2G110145*); chromosome Bd3 for *Brachypodium* (*BRADI_3g16307v3*); chromosome 8 for rice (*LOC_Os08g06380*) (Table 1).

**Figure 2:**
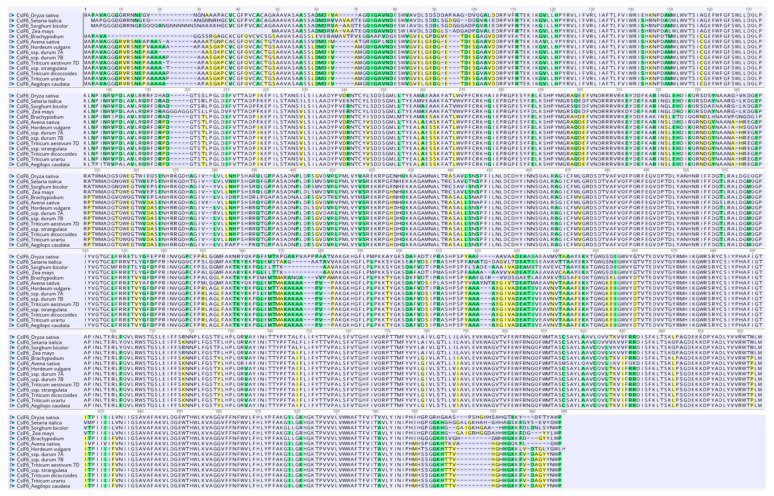
Plant CslF6 amino acid sequences for the mature protein are aligned with Clustal V: *Ae. caudata* (present report), wheat (A, B and D genomes), *T. urartu* (*XP_048543309.1*), *T. dicoccoides* (*XP_037457806.1*), *Brachypodium* (*XP_003573454.1*), rice (*AKJ66179.1*), maize (*AKJ66177.1*), *Setaria* (*XP_004972774.1*), *Sorghum* (*XP_002445102.1*), *Avena* (*AKJ66176.1*), barley (*ABZ01578.1*), *Ae. Stranguata* (*XP_020187382.1*).

The newly obtained sequence was used for the determination of the amino acid sequence through Geneious (version 2023.2.1) software (Figure 2). 

### 3.2. Comparison of Amino Acid Sequences from Other Species

Once the *Ae. caudata CslF6* gene sequence was isolated, a protein structure prediction was performed to define the differences among a selection of monocot sequences available in public databases and relate them to the final β-glucan content and DP3/DP4 ratio in the different species. The DP3 and DP4 represent two major oligomer units obtained from the enzymatic digestion of the β-glucan, which explain more than 90% of cereal β-glucan structures and are strongly correlated to the degree of solubility of the polysaccharide [43].

**Figure 3:**
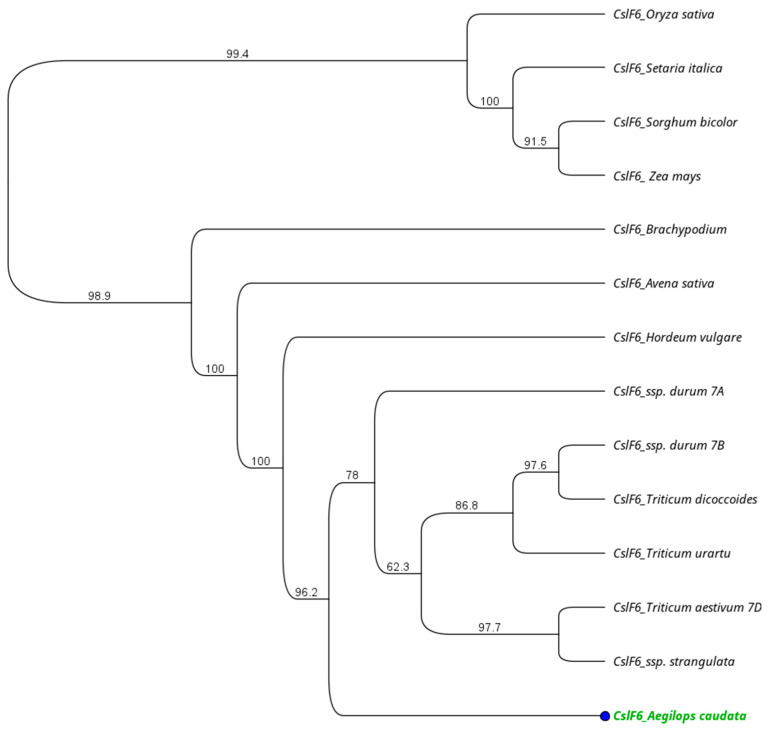
Phylogenetic relationships among the CslF6 polypeptide from *Ae. caudata* (green text), wheat (A, B and D genomes), *T. urartu*, *T. dicoccoides*, *Brachypodium*, rice, maize, *Setaria*, *Sorghum*, avena, barley, *Ae. Stranguata*.

The closest match was found with wheat genome D and *Aegilops taushii* ssp. *strangulata* (98%), followed by the other *Triticum* subspecies (~98%) and barley (97%). The more divergent sequences were the CslF6 from *Sorghum* and *Setaria*, at 81.4% and 81.6%, respectively. The amino acid length for each species differed, with some of them being of identical length. In order to investigate the evolutionary distances, the UPGMA tree was implemented in Geneious (version 2023.2.1) software. The tree showed two main clusters, one including the CslF6 protein sequences from rice, maize, sorghum, and *Setaria*, and a second one with all the other monocot species considered. Among the second group, the Triticum subspecies clustered all together, while *Brachypodium*, oat, and barley grouped independently. In the evolutionary tree, the *Ae. caudata* gene represents the outline of the *Triticum* cluster (Figure 3).

### 3.3. Amino Acid Sequence, (1,3;1,4)-β-Glucan Amounts and DP3:DP4 Ratios

**Figure 4:**
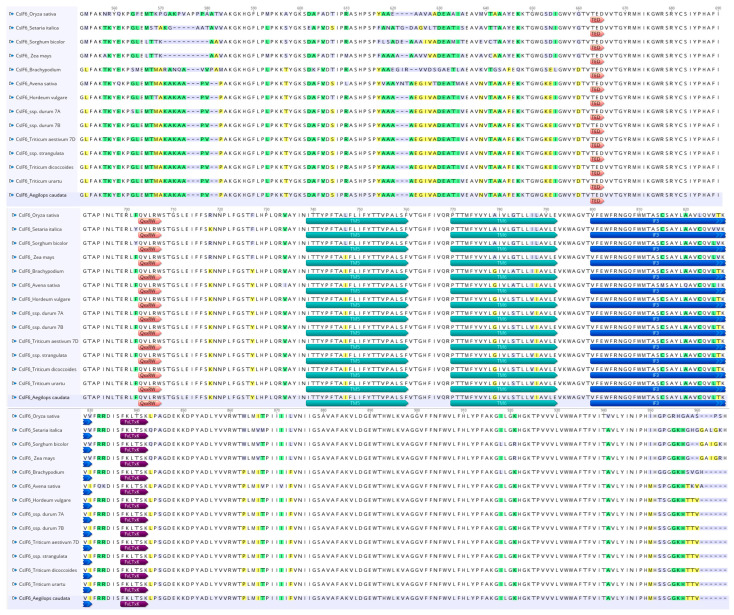
Comparison of CslF6 protein sequences from a selection of plants with colored residues highlighting sequence discrepancies. White boxes indicate a similarity of 100%, green boxes a similarity from 80% to 100%, yellow boxes a similarity from 60 to 80%, and purple boxes a similarity less than 60%. Shown are important predicted trans-membrane helices (TM5 and TM6), the FxLTxK motif, and the TED and QxxRW motifs. The cytoplasmic amphipathic helix (IF3) is also shown.

The amino acid sequences of the monocot sequences considered are highly conserved, and their 3D structures are likely to exhibit similarities (Figure 4). Considering that the amino acid sequence is of fundamental importance for the protein structure and β-glucan biosynthesis, a comparative analysis was carried out on the principal motifs using the homology model based on the *Rhodobacter sphaeroides* BCSA cellulose synthase subunit crystal structure [44].

As shown in Figure 4, the *Ae. caudata* protein sequence, the similarity in amino acid sequences between CslF6 enzymes from *Brachypodium*, barley, wheat, *T. dicoccoides*, *T. urartu* and *Ae. strangulata* suggests resemblances in their 3D structures.

Figure 4 showed that the G/D residue sits just before the anticipated “finger helix,” adjacent to the TED motif believed to interact with the nascent polysaccharide’s acceptor end. 

Additionally, two other residues are reported on Figure 4, the W residue in the conserved QxxRW motif highlighting the catalytic pocket at the membrane distal side, which coordinates the translocation and elongation of the glucosidic units in the β-glucan biosynthesis, and the Y/F residue near the core QxxRW catalytic motif that interacts with CslF6’s putative gating loop, housing an FxLTxK motif (Figure 4).

Both the differences highlighted in the residues close to the TED and QxxRW motif have an effect on contributing to the difference in the DP3:DP4 ratio [31].

### 3.4. Analysis of Cis-Acting Elements in CslF6 Promoter Region of Ae. caudata Gene

**Figure 5:**
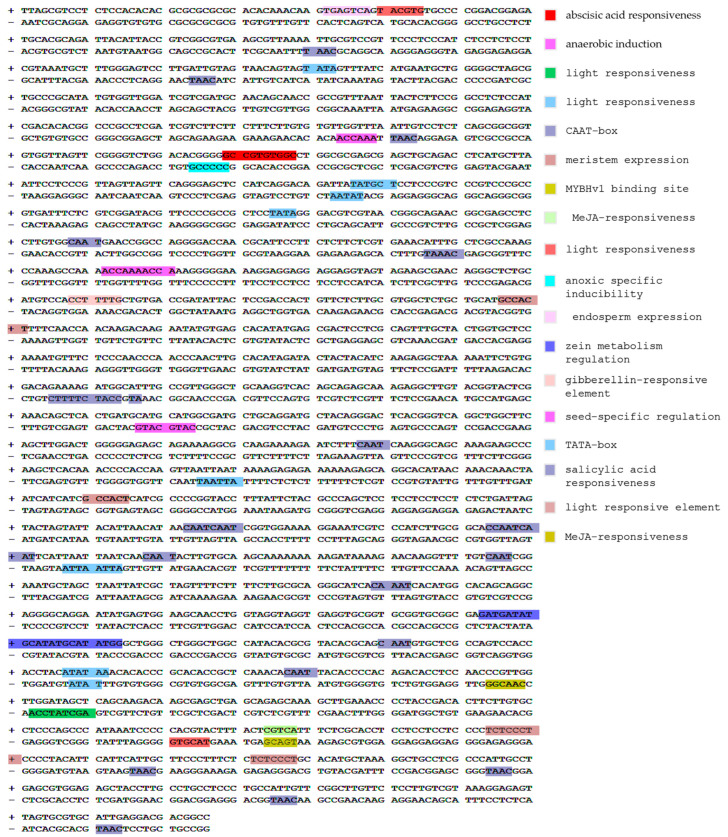
Cis-acting regulatory element detected in the promoter sequence of the *CslF6* gene in *Ae. caudata*.

The gene expression pattern primarily relies on cis-acting elements found within the regulatory regions of a promoter. Prediction of cis-acting elements of the *CslF6* gene promoter of *Ae. caudata* revealed a total of 18 variations (depicted in Figure 5). These elements encompassed a diverse range, including light-responsive (G-box, TCCC-motif and ATC-motif), defense and stress-related (ARE-element), phytohormone-responsive (ABRE), and growth and development-associated elements (CAT-box, GCN4-motif, MSA-like, O2-site and RY-element). Notably, G-box, ARE-element, TGACG-motif, and CGTCA-motif were prevalent, suggesting their pivotal role in stress resilience, growth, and development. Additionally, the gene appeared to be significantly regulated by cis-regulatory elements found to be involved in plant hormone responsiveness, including gibberellins, salicylic acid responsiveness and jasmonic acid. 

## 4. Discussion

The isolation and characterization of the *cellulose synthase-like F6* gene (*CslF6*) in *Ae. caudata* have unveiled significant insights into the molecular architecture and functional implications of this gene within the species. Our study employed a multifaceted approach, integrating genomic sequence analysis, protein structure prediction, and comparative genomics to elucidate the distinct attributes of *CslF6* in *Ae. caudata*.

One key aspect, unveiled through this study, is the structural and functional conservation observed across related species. The high sequence identities between the CslF6 gene of *A. caudata* and wheat genomes (A, B, and D) underscore evolutionary links among these species. The conservation of intron/exon structures across monocots like durum wheat, maize, and oat, echoing that of *Aegilops caudata*, signifies an underlying genetic coherence in β-glucan biosynthesis among these cereals, useful for new breeding programs

We obtained the complete sequence of the *CslF6* gene in *Ae. caudata*, and we compared our data with *CslF6* sequences from other species. The gene had the same intron/exon structure as durum wheat, maize, and oats, including three exons and two introns [42,45,46].

Comparative analyses, particularly in the context of genomic and cDNA similarities with wheat genomes, underscored high identities between the *Ae. caudata CslF6* and the A, B, and D genomes of wheat, signifying evolutionary relationships and conservation across these species. Furthermore, protein structure predictions and comparative assessments with a spectrum of monocot sequences elucidated pivotal similarities and divergences. Notably, the closest matches were identified with the D wheat genome and *Ae. tauschii* ssp. *strangulata*, implying a close evolutionary association among these genomes. The results are strongly correlated to the genome’s evolution; in fact, the ancestor of the bread wheat D genome is *Ae. tauschii* (Coss.) [47]. Additionally, we located the gene on chromosome 7C of the *Ae. caudata* genome, and we made a comparison with the location in other monocots. The synteny observed in the map locations suggested that these genes were highly conserved, as already reported by previous authors [48].

The investigation into amino acid sequences from various monocot species revealed significant conservation, suggesting analogous 3D structures among these CslF6 enzymes. Crucially, specific motifs within the amino acid sequences were pinpointed, notably the monocots used for the comparative analysis, which showed differences in the residue near the ‘finger helix’ (G/D) and the residue (Y/F) adjacent to the core QxxRW catalytic motif. No differences were underlined in the W residue within the conserved QxxRW motif. These residues are implicated in critical interactions in β-glucan biosynthesis, particularly influencing the DP3:DP4 ratio, thereby potentially affecting the final β-glucan content [31,44].

The observed variations in these residues, specifically those proximal to the TED and QxxRW motifs, are likely contributors to differences in the DP3:DP4 ratio, aligning with existing literature [31,44] and demonstrating their impact on β-glucan composition. This highlights the functional relevance of specific amino acid residues in shaping the enzymatic activity and structural features of CslF6, ultimately influencing the β-glucan synthesis pathway [33,44].

Moreover, the placement of *Ae. caudata* within an evolutionary tree, particularly as an outlier within the *Triticum* cluster, underscores its distinctiveness within the context of *CslF6* evolution among monocots, signifying potential unique functional adaptations or genetic divergence within this species as previously reported in literature [49].

The analysis of the cis-acting elements in the promotor region of the *CslF6* gene in the *Ae. caudata* genome highlighted the presence of many motifs associated with plant growth and development, stress response, and hormone regulation. These results suggested that the *CslF6* gene family may participate in plant growth and development as well as stress tolerance, and the family members are regulated by plant hormones as already reported in other species [50,51,52].

The potential applications of these findings extend to breeding strategies aimed at enhancing cereal varieties’ nutritional content and resilience. Harnessing the genetic resources within *Ae. caudata* for targeted breeding or employing genetic modification techniques holds promise for developing cereals with improved β-glucan content, balanced amino acid profiles, and enhanced micronutrient richness. Moreover, this knowledge aids in creating crops resilient to environmental stresses, thereby contributing to sustainable agricultural practices.

Furthermore, the detailed characterization of the *CslF6* gene and its protein structure in *Ae. caudata* provides a foundational framework for future studies. Exploring the regulatory mechanisms underlying β-glucan biosynthesis, investigating additional genetic factors influencing cereal nutritional content, and conducting functional validations of specific amino acid residues could offer deeper insights into refining cereal traits for human consumption.

## 5. Conclusions

The present work focuses on the cellulose synthase-like F6 gene (CslF6) in the *Ae. caudata* genome, providing valuable insights into the molecular processes of β-glucan biosynthesis and their potential impact on cereal nutrition. Utilizing genomic analysis, protein structure prediction, and comparative genomics, the research uncovers unique characteristics of CslF6 in *Ae. caudata*.

Comparative analysis of CslF6 amino acid sequences across monocot species reveals significant conservation, suggesting similar 3D structures. However, crucial motifs near the 'finger helix' and the QxxRW catalytic motif exhibit variations among species, influencing the DP3:DP4 ratio and, consequently, β-glucan content in cereals. These variations emphasize the role of specific residues in shaping CslF6 enzymatic activity and structural features.

The placement of *Ae. caudata* as an outlier in the evolutionary tree within the Triticum cluster signifies its distinctiveness in CslF6 evolution among monocots, hinting at potential unique functional adaptations or genetic divergence.

Exploring the promoter region of the CslF6 gene in the Ae. caudata genome reveals cis-acting elements associated with essential processes like plant growth, stress response, and hormone regulation. This implies multifaceted functions for the CslF6 gene family. The observed regulation by plant hormones aligns with known mechanisms in other species, highlighting evolutionary conservation.

In conclusion, this research deepens our understanding of β-glucan biosynthesis and suggests leveraging *Ae. caudata*’s genetic resources for improving cereal nutrition through breeding or genetic modification. The study’s insights pave the way for further exploration and exploitation of these genetic resources in crop improvement programs focused on sustainable and nutritious food production.

## Figures and Tables

**Table 1:** Chromosome location of CslF6 gene in a set of monocotyledons used for the analysis. For each species, the accession number relative to the sequence was reported.

Species	Chromosome Location	Accession Number
*H. vulgare*	7H	HORVU7Hr1G070010
*T. turgidum* ssp. *Durum*	7A	TRITD7Av1G149750
	7B	TRITD7Bv1G108090
*T. aestivum*	7A	TraesCS7A02G298600
	7B	TraesCS7B02G188400
	7D	TraesCS7D02G294300
*T. dicoccoides*	7A	TRIDC7AG041550
	7B	TRIDC7BG030910
*S. bicolor*	7	SORBI_3007G050600
*A. sativa*	7A	AVESA.00001b.r3.7Ag0002427
	7C	AVESA.00001b.r3.7Cg0002511
	7D	AVESA.00001b.r3.7Dg0001419
*T. urartu*	7A	LOC125522276
*A. strangulata*	7D	LOC109773098
*S. italica*	6	XM_004972717
*Z. mays*	10	GRMZM2G110145
*Brachypodium*	Bd3	BRADI_3g16307v3
*O. sativa*	8	LOC_Os08g06380

## Data Availability

The data presented in this study are available on request from the corresponding author. The data are not publicly available due to privacy.

## Abbreviations

Ces, cellulose synthase; Csl, cellulose-synthase-like; DP3, 3-O-β-cellobiosyl-D-glucose; DP4, 3-O-β-cellotriosyl-D-glucose; BCSA, bacterial cellulose synthase A; TED motif, threonine-glutamic acid-aspartic acid motif; QxxRW motif, binding site for the terminal disaccharide of the glucan; TM5, trans-membrane helix 5; TM6, trans-membrane helix 6; IF3, cytoplasmic amphipathic helix; G-box, CACGTG box; TCCC-motif, element involved in defense and stress responsiveness; ATC-motif, conserved DNA module involved in light responsiveness; ARE-element, adenylate-uridylate-rich elements; ABRE, ABA response elements; CAT-box, element related to meristem expression; GCN4-motif, ‘leucine-zipper’ recognition; MSA-like, mitosis-specific activator; O2-site, cis-acting regulatory element involved in zein metabolism regulation; RY-element, element involved in seed-specific regulation.
